# Supplementary material for: Altruistic disease signalling in ant colonies
Source: Nat Commun. 2025 Dec 2;16:10511. doi: 10.1038/s41467-025-66175-z (PMC12672639; doi:10.1038/s41467-025-66175-z)
Supplement: Supplementary file 1 — Supplementary Information [file 41467_2025_66175_MOESM1_ESM.pdf]

Supplementary Information for:

Altruistic disease signalling in ant colonies

Dawson et al.

This file contains:

Supplementary Tables S1-S15

Supplementary Figures S1-S6

Supplementary References

Supplementary Tables

Supplementary Table S1. Effect of pupal infection and worker presence on worker pupae CHCs.

(a) Statistical results for each of the four CHC peaks identified by Pull et al 2018[1] as possible unpacking cue or signal in *L. neglectus* worker pupae, containing the two immune-associated peaks C33:2 and C33:1, as well as the peaks of C35:2, and co-eluting C35:2 + C35:1. For the relative abundance of each peak, we used a Linear Model (LM) to test for a significant interaction between the main effects of pupal infection and the presence/absence of tending workers, if the overall model was significant. Sample size of total 323 worker pupae, of which n = 69 infected without workers (I+W-), n = 133 infected with workers (I+W+), n = 64 uninfected controls without workers (I-W-) and n = 57 uninfected controls with workers (I-W+). **(b)** Following the significant interactions found for C33:2 and C33:1 we performed a linear model (LM) per peak to test for a significant effect of infection load on peak abundance, controlling for population, infection period and worker presence. The analysis focuses only on fungus-infected pupae (n = 201, since PCR could not be conducted on one sample). For both (a) and (b), the data were transformed using the ordered quantile normalisation prior to analyses. Model details, peak (Table S13), test statistic, degrees of freedom (df), p-value and effect size given. Exact, two-sided p-values adjusted for multiple testing (as detailed in the Methods) are reported. Significant p values highlighted in bold, "-" indicates non-applicable due to non-significance. Note for (b) that already the uncorrected raw p-values were > 0.107, hence the absence of significance was not induced by correction for multiple testing.

a)

Relative peak abundance – worker pupae								
LM	peak	overall model			interaction			
		χ^2	df	p-value	χ^2	df	p-value	effect size (η^2p)
**abundance ~ infection treatment * worker presence** + population + infection period	C33:2	32.812	3	**1.4×10^{-6}**	7.044	1	**0.0080**	0.02
	C33:1	17.248	3	**0.0008**	4.419	1	**0.0356**	0.01
	C35:2	18.571	3	**0.0007**	0.763	1	0.382	-
	C35:2+ C35:1	3.759	3	0.289	-	-	-	-

b)

Effect of infection load on peak abundance – worker pupae				
LM	peak	overall model		
		χ^2	df	p-value
**abundance ~ infection load** + population + infection period + worker presence	C33:2	1.939	1	0.164
	C33:1	2.598	1	0.164

Supplementary Table S2. Statistical results of comparison of the four worker pupae groups based on their immune-associated CHCs. Linear Mixed Model (LMM) results testing the differences between the four treatment groups, i.e. infected worker pupae without workers (I+W-), infected worker pupae with workers (I+W+), control worker pupae without infection and without workers (I-W-) and control worker pupae with workers (I-W+), based on both C33:2 and C33:1. Sample sizes as in Table S1. Data were transformed using the ordered quantile normalisation prior to analyses. Model details, test statistic, degrees of freedom (df) and p-value for the overall model and all-pairwise post hoc comparisons are given. Exact, two-sided post hoc p-values adjusted for multiple testing are reported. Significant p-values highlighted in bold and shown by letters a and b for the two significance groups (I+W+ vs all others) in Fig. 1a. "-" indicates non-applicable due to non-significance.

Relative abundance of combined immune-associated peaks - worker pupae										
LMM	peaks	overall model			interaction			pairwise comparisons		
		χ^2	df	p-value	χ^2	df	p-value	Pair	p-value	effect size (Cohen's d)
**abundance ~ infection treatment * worker presence** + peak+ population + infection period + (1|individual)	C33:2 and C33:1	16.554	3	**0.00087**	4.575	1	**0.0324**	I+W+ vs I+W-	**0.0001**	0.778
								I+W+ vs I-W+	**0.012**	0.527
								I+W+ vs I-W-	**0.002**	0.623
								I+W- vs I-W-	0.535	-
								I+W- vs I-W+	0.352	-
								I-W- vs I-W+	0.657	-

Supplementary Table S3. Effect of pupal infection and worker presence on immune gene expression of worker pupae. Statistical results for the three candidate immune genes (*BGBP*, *PGRP-SC2* and *Def1*) for the worker pupae. For the expression levels of **(a)** each individual gene and **(b)** a combined immune value (mean value per pupa after z-transformation per gene), we used a Linear Model (LM) to test for a significant interaction between the main effects of infection treatment and the presence/absence of tending workers, if the overall model was significant. As the interaction was not significant for any of the models, we then tested for a significant effect of the main effects of worker presence and infection treatment. The sample size totalled 117 worker pupae, of which n = 19 infected without workers (I+W-), n = 58 infected with workers (I+W+), n = 20 uninfected controls without workers (I-W-) and n = 20 uninfected controls with workers (I-W+). Data were transformed using the ordered quantile normalisation prior to analysis. Model details, test statistic, degrees of freedom (df) and p-value for the overall model, interaction between the two main effects and the main effects individually. Exact, two-sided p-values adjusted for multiple testing are reported, and shown in bold when significant.

a)

Individual gene expression levels – worker pupae														
LM	immune gene	overall model			interaction			worker presence			treatment			
		χ^2	df	p-value	χ^2	df	p-value	χ^2	df	p-value	χ^2	df	p-value	effect size (η^2p)
**expression level ~ infection treatment * worker presence** + infection period	*BGBP*	68.844	3	**7.5×10^{-13}**	2.528	1	0.112	0.015	1	0.902	63.713	1	**1.4×10^{-15}**	0.45
	PGRP-SC2	83.072	3	**3.3×10^{-16}**	0.019	1	0.890	2.065	1	0.151	74.918	1	**2.2×10^{-16}**	0.51
	Def1	88.766	3	**3.3×10^{-16}**	0.004	1	0.951	0.006	1	0.939	85.376	1	**2.2×10^{-16}**	0.53

b)

Combined gene expression levels – worker pupae														
LM	overall model			interaction			worker presence			treatment				
	χ^2	df	p-value	χ^2	df	p-value	χ^2	df	p-value	χ^2	df	p-value	effect size (η^2p)
**expression level ~ infection treatment * worker presence** + infection period	83.568	3	**2.2×10^{-16}**	0.313	1	0.576	0.530	1	0.467	77.698	1	**2.2×10^{-16}**	0.48

Supplementary Table S4. Effect of pupal infection and worker presence on queen pupae CHCs. (a) Statistical results for the four candidate CHC peaks for the queen pupae (as in Table S1a for the worker pupae). For none of the four peaks, its relative abundance was found to significantly differ in the overall LM, so that no further statistics were performed. Sample size of a total of 103 queen pupae, of which n = 19 infected without workers (I+W-), n = 45 infected with workers (I+W+), n = 19 uninfected controls without workers (I-W-) and n = 20 uninfected controls with workers (I-W+). **(b)** As for the worker pupae (Table S1b) we also performed a linear model (LM) each to test for an effect of infection load on abundance of the two immune-associated peaks (C33:2 and C33:1), controlling for infection period and worker presence/absence. Analysis carried out on fungus-infected pupae only (n = 64). To obtain normality of data distributions, data for each peak were transformed prior to analyses using the following transformations: C33:2 box-cox transformed, C33:1 log transformed, C35:2 square root transformed; C35:2+C35:1 ordered quantile normalisation, and infection load data were transformed using the ordered quantile normalisation. Model details, peak (Table S13), test statistic, degrees of freedom (df), and p-value given. Exact, two-sided p-values adjusted for multiple testing are reported. Note that also the uncorrected raw p-values were > 0.472 in all of the peaks in (a) and > 0.534 in (b), hence the absence of significance was not driven by multiple model comparisons.

a)

Relative peak abundance – queen pupae				
LM	peak	overall model		
		χ^2	df	p-value
**abundance ~ infection treatment * worker presence** + infection period	C33:2	1.789	3	0.816
	C33:1	1.044	3	0.816
	C35:2	2.516	3	0.816
	C35:2+C35:1	0.940	3	0.816

b)

Effect of infection load on peak abundance – queen pupae				
LM	peak	overall model		
**abundance ~ infection load** + infection period + worker presence		χ^2	df	p-value
	C33:2	0.0309	1	0.837
	C33:1	0.0426	1	0.837

Supplementary Table S5. Statistical results of comparison of the four queen pupae groups based on their immune-associated CHCs. Linear Mixed Model (LMM) results testing the differences between the four treatment groups, i.e. infected or uninfected queen pupae, each with or without workers, based on C33:2 and C33:1. Sample sizes as in Table S4. To obtain normality, data were transformed using the Yeo-Johnson transformation. Model details, test statistic, degrees of freedom (df) and exact, two-sided p-values are given. Since the overall model was non-significant, indicated by ns in Fig. 1B, no pairwise post hoc comparisons were calculated.

**Relative abundance of combined immune-associated peaks in the queen pupae**				
LMM	peaks	overall model		
		χ^2	df	p-value
**abundance ~ infection treatment * worker presence** + peak + infection period + (1|individual)	C33:2 and C33:1	2.736	3	0.434

Supplementary Table S6. Statistical testing for a potential signalling of queen pupae via the non-candidate CHC peaks. To test whether queen pupae may use different peaks than the candidate peaks identified by Pull et al. 2018[1] for the worker pupae for possible disease signalling, we also tested the remaining 14 peaks of the pupal chemical profile (for which quantification of their ^{12}C and ^{13}C proportions was possible for both worker and pupae; see Methods), for a possible difference according to infection treatment and worker presence. Sample sizes as in Table S4. To obtain normality of data distributions, data for each peak were transformed prior to analyses using the following transformations: C27, C28, C29, C33+13MeC33:1, C34+14MeC34:1+12MeC34:1, C35+13MeC35:1, C37+13MeC37:1 ordered quantile normalisation; 3MeC29, C30, C31, 3MeC33 log transformation; C33:1 (non-candidate C33:1 with RI 3289; Table S13), 13MeC33 box-cox transformation; 3MeC33:1 arcsinh transformation. Model details, peak (Table S13), test statistic, degrees of freedom (df) and exact, two-sided p-value after correction for multiple testing (as detailed in the Methods) given. None of the overall models were significant after p-value adjustment for multiple testing. Note that three CHCs (C27, 13MeC33 and 3MeC33) were significant in the overall model in the absence of adjustment, yet their interaction terms were all > 0.43, therefore supporting that we found no evidence of chemical signalling in the queen pupae.

**Relative abundance of non-candidate peaks in the queen pupal profile**				
LM	peak	overall model		
		χ^2	df	p-value
**abundance ~ infection treatment * worker presence** + infection period	C27	9.74	3	0.141
	C28	4.38	3	0.474
	C29	3.202	3	0.474
	3MeC29	5.100	3	0.461
	C30	3.596	3	0.474
	C31	2.703	3	0.474
	C33:1	1.988	3	0.575
	C33+13MeC33:1	3.560	3	0.474
	13MeC33	8.935	3	0.141
	3MeC33:1	5.759	3	0.434
	3MeC33	10.908	3	0.141
	C34+14MeC34:1 +12MeC34:1	3.764	3	0.474
	C35+13MeC35:1	3.067	3	0.474
	C37+13MeC37:1	2.792	3	0.474

Supplementary Table S7. Worker unpacking behaviour directed to infected vs uninfected pupae. Statistical results for the cox proportional-hazards regression of the effect of infection on unpacking behaviour. Infected worker pupae elicited significantly more unpacking by the workers than uninfected controls, whilst there was no significant effect of infection treatment in the queen pupae. Model, test statistic, degrees of freedom (df), and p-value given. For the queen pupae, the overall model directly gives the effect of treatment, as all stem from the same population. For the significant effect of treatment (infected, uninfected) in the worker pupae, the hazard ratio of being unpacked when infected and its CI are reported ("-" for queen pupae due to non-significance). Exact, two-sided p-values given, and shown in bold when significant. Samples sizes as in Tables S1a, S4a.

**Worker unpacking towards worker resp. queen pupae**								
pupal caste	Cox proportional-hazards regression	overall model			infection treatment		
		Wald test	df	p-value	p-value	HR	CI
**worker**	**unpacking ~ infection treatment** + population	8.82	2	**0.010**	**0.0037**	4.542	1.64-12.60
**queen**	**unpacking ~ infection treatment**	2.49	1	0.115	equal to overall	-	-

Supplementary Table S8. Effect of pupal infection and worker presence on immune gene expression of queen pupae. Statistical results for the three candidate immune genes (*BGBP*, *PGRP-SC2* and *Def1*) for the queen pupae. For the expression levels of **(a)** each individual gene and **(b)** a combined immune value (mean value per pupa after z-transformation per gene), we used a Linear Model (LM) to test for a significant interaction between the main effects of infection treatment and the presence/absence of tending workers, if the overall model was significant. As the interaction was not significant for any of the genes, we then tested for a significant effect of worker presence and infection treatment. Total sample size of 62 queen pupae, of which n = 17 infected without workers (I+W-), n = 20 infected with workers (I+W+), n = 11 uninfected controls without workers (I-W-) and n = 14 uninfected controls with workers (I-W+). Data were transformed using the ordered quantile normalisation prior to analysis. Model details, test statistic, degrees of freedom (df) and p-value for the overall model, interaction between the two main effects and the main effects individually. Exact, two-sided p-values adjusted for multiple testing at the model level are reported, and shown in bold when significant.

a)

**Individual gene expression levels – queen pupae**														
LM	immune gene	overall model			interaction			worker presence			treatment			
		χ^2	df	p-value	χ^2	df	p-value	χ^2	df	p-value	χ^2	df	p-value	effect size (η^2p)
**expression level ~ infection treatment * worker presence** + infection period	*BGBP*	16.211	3	**0.0010**	0.012	1	0.914	2.080	1	0.149	14.718	1	**0.00013**	0.21
	PGRP-SC2	18.258	3	**0.0006**	1.908	1	0.167	0.061	1	0.805	16.328	1	**5.3×10^{-5}**	0.23
	Def1	19.516	3	**0.0006**	1.082	1	0.298	1.377	1	0.241	17.535	1	**2.8×10^{-5}**	0.24

b)

Combined gene expression levels – queen pupae													
LM	overall model			interaction			worker presence			treatment			
	χ^2	df	p-value	χ^2	df	p-value	χ^2	df	p-value	χ^2	df	p-value	effect size (η^2p)
**expression level ~ infection treatment * worker presence** + infection period	18.826	3	**0.0003**	0.572	1	0.449	0.918	1	0.338	17.677	1	**2.6×10^{-5}**	0.25

Supplementary Table S9. Infection load timeline in worker pupae and queen pupae. Statistical results for the comparison in fungal infection loads of **(a)** worker pupae and **(b)** queen pupae at the different periods in infection progression from the start of the experiment (which began three days after pathogen exposure). Samples for the 'early' infection period were collected at 6 & 12 hours, for the 'middle' at 18 & 24 hours and for the 'late' at 30, 36 & 42 hours after the start of the experiment. Sample sizes for the worker pupae were n = 75 early, n = 57 middle and n = 69 late infection period samples, whilst n = 16 early, n = 16 middle and n = 32 late for the queen pupae. To obtain normality of data distribution, data were transformed prior to analyses using the ordered quantile normalisation. Model details, test statistic, degrees of freedom and p-value given. Pairwise tests between infection periods were corrected for multiple testing; effect sizes given for significant effects. "-" indicates non-applicable due to non-significance. Exact, two-sided p-values reported, significant p-values shown in bold.

a)

**Infection progress – worker pupae**						
LM	overall model			pairwise comparisons		
	χ^2	df	p-value	pair	p-value	effect size (Cohen's d)
**infection load ~ infection period** + population + worker presence	36.997	2	**9.3×10^{-9}**	early to middle	**0.016**	0.441
				middle to late	**0.0007**	0.643
				early to late	**7.2×10^{-9}**	1.084

b)

**Infection progress – queen pupae**						
LM	overall model			pairwise comparisons		
	χ^2	df	p-value	pair	p-value	effect size (Cohen's d)
**infection load ~ infection period** + worker presence	10.178	2	**0.006**	early to middle	0.161	-
				middle to late	**0.006**	-1.002
				early to late	0.180	-

Supplementary Table S10. Unpacking of healthy pupae after application of signal vs. non-signal extract. Unpacking of healthy worker resp. queen pupae in a bioassay testing whether the CHC extract of signalling worker pupae would elicit higher worker unpacking than the extract of non-signalling control pupae. Statistical results for the binomial Generalised Linear Models (GLM) of the effect of extract type (signal vs non-signal) on the unpacking of the treated, healthy worker and queen pupae. For worker pupae we controlled for population, experimental block and batch while for the queen pupae, which originated from a single population, we controlled for experimental block and batch only. One-sided p-values are reported.

pupal caste	binomial GLM	extract type		
		χ^2	df	p-value
**worker**	**unpacking ~ extract type** + population + experimental block + experimental batch	2.717	1	**0.0496**
**queen**	**unpacking ~ extract type** + experimental block + experimental batch	4.768	1	**0.0145**

Supplementary Table S11. Identified isomers of the C33:2 signal peak and their relative abundances in signal vs. non-signal extract. Positional di-unsaturated C33 isomers (X,Y-C33:2) as identified from the pupal extracts after DMDS-derivatisation and GC-MS analysis of the DMDS-di-adducts, using the ion pairs $(A)^+$ and $(D)^+$ as diagnostic fragments (SIM data; following Carlson et al. 1989[2]). Kováts Retention Index (RI) and both diagnostic ions provided for each DMDS-di-adduct. The normalised EIC-abundance of the $(A)^+$ fragment per extract was calculated as the proportion of the $(A)^+$ abundance of each isomer on the total $(A)^+$ abundance of all detected isomers of the respective extract. For each isomer, we provide the ratio of its relative abundance in the signal / non-signal extract, with values > 1 indicating that the isomer is overrepresented in relative abundance in the signal extract compared to the non-signal extract, and *vice versa* for values < 1. Note that out of the eight isomers identified in the signal extract, only the $(A)^+$ ion was unambiguously detectable for three isomers in the non-signal extract (12,20-C33:2; 9,15-C33:2; and 7,21-C33:2; indicated by§), likely due to the $(D)^+$ ion signal being obscured by the elevated baseline for these low-abundant peaks. However, they could be confirmed by the SIM data for 12,20-C33:2 and 7,21-C33:2. Moreover, the $(A)^+$ to $(D)^+$ ion ratio could not be reliably resolved for 10,22-C33:2 in the non-signal extract, likely due to co-elution with other fragments, which lead to an overestimation of the isomer's relative abundance in the non-signal extract, indicated by setting the ratio in italics. Absolute abundances of the DMDS-di-adducts were approx. 20-times lower in the non-signal than in the signal extract.

		ion pair (*m/z*)		normalised $(A)^+$ abundances		ratio
X,Y-C33:2	RI	$(A)^+$	$(D)^+$	signal extract	non-signal extract	signal / non-signal extract
12,20-	4753	215.2	229.2§	0.043	0.039	1.10
11,21-	4767	201.1	215.2	0.346	0.314	1.10
10,22-	4774	187.1	201.1	0.087	0.154	*0.57*
10,20-	4769	187.1	229.2	0.232	0.214	1.08
9,23-	4785	173.1	187.1	0.088	0.115	0.76
9,21-	4779	173.1	215.2	0.083	0.066	1.27
9,15-	4739	173.1	299.2§	0.057	0.047	1.21
7,21-	4797	145.1	215.2§	0.064	0.050	1.28

Supplementary Table S12. Identified isomers of the C33:1 signal peak and their relative abundance in signal vs. non-signal extract. Positional mono-unsaturated C33 isomers (X-C33:1) identified, as above for the di-unsaturated isomers (Table S11), yet using the ion pairs $(A)^+$ and $(B)^+$ as diagnostic fragments. Kováts Retention Index (RI) and both diagnostic ions provided for each DMDS-mono-adduct. The normalised EIC-abundance of the $(A)^+$ fragment per extract was calculated as the proportion of the $(A)^+$ abundance of each isomer on the total $(A)^+$ abundance of the detected isomers of the 13- to 9-isomer cluster (native C33:1 peak with RI 3281, Table S13) respective extract (whereas the 7-C33:1 isomer constitutes a separate C33:1 peak of RI 3289, which is not related to unpacking[1], Table S13, Methods). For each isomer of the signal peak C33:1 (RI 3281), we provide the ratio of its relative abundance in the signal / non-signal extract, with values > 1 indicating that the isomer is overrepresented in the signal extract compared to the non-signal extract, and *vice versa* for values < 1. Both diagnostic ions for each isomer were detected via EIC in both the signal and non-signal extract, as the isomer concentrations were sufficiently high for a reliable GC-MS analysis in both extract types. Absolute abundances of the DMDS-mono-adducts were approx. 3-times lower in the non-signal than in the signal extract.

		ion pair (*m/z*)		normalised $(A)^+$ abundances		ratio
X-C33:1	RI	$(A)^+$	$(B)^+$	signal extract	non-signal extract	signal / non-signal extract
13-	3965	229.2	327.3	0.160	0.085	1.89
12-	3970	215.2	341.3	0.307	0.502	0.61
11-	3968	201.2	355.4	0.176	0.092	1.91
10-	3972	187.1	369.4	0.075	0.075	1.00
9-	3975	173.1	383.4	0.281	0.246	1.14

Supplementary Table S13. Cuticular hydrocarbons (CHCs) in *Lasius neglectus* worker and queen pupae. The 18 CHCs for which quantification of their ^{12}C and ^{13}C proportions was possible for both worker and queen pupae (see Methods), their chain length and structure, as well as their Kováts Retention Index, RI (averaged RI based on a representative subset of 54 worker pupae processed in the same GC-MS run), are given. CHC identification followed Pull et al. 2018[1], Ugelvig et al. 2008[3] and Cremer et al. 2008[4]. Note that the peaks with RIs 3302, 3403 and 3698 were dominated by the *n*-alkanes C33, C34 resp. C37 in Pull et al. 2018, while in the current study, methyl-branched alkenes identified in Cremer et al. 2008 for the workers became increasingly dominant within these peaks, with the effect becoming stronger with increasing CHC chain length. Co-eluting CHCs are listed in the table, with the dominant given in italics. Mass spectra representing the dominant compounds per peak are deposited under doi.org/10.15479/AT-ISTA-20471. Candidate peaks that have been identified as showing higher abundance in worker pupae that were destructively disinfected by the workers due to infection with the fungal pathogen *Metarhizium brunneum* by Pull et al. 2018[1] shown in bold – note that this only applies to the first (RI 3281) of the two separate C33:1 peaks. All peaks are present in both worker and queen pupae.

CHC names	CHC chain length and structure	RI
n-Heptacosane	C27	2700
n-Octacosane	C28	2800
n-Nonacosane	C29	2900
3-Methylnonacosane	3MeC29	2975
n-Triacontane	C30	3000
n-Hentriacontane	C31	3100
**Tritriacontadiene**	**C33:2**	**3254**
**Tritriacontene**	**C33:1**	**3281**
Tritriacontene	C33:1	3289
n-Tritriacontane<br>*+ 13-Methyltritriacontene*	C33+*13MeC33:1*	3302
13-Methyltritriacontane	13MeC33	3327
3-Methyltritriacontene	3MeC33:1	3352
3-Methyltritriacontane	3MeC33	3374
n-Tetratriacontane<br>*+14-Methyltetratriacontene*<br>*+12-Methyltetratriacontene*	C34+*14MeC34:1+12MeC34:1*	3403
**Pentatriacontadiene**	**C35:2**	**3452**
**Pentatriacontadiene**<br>**+ *Pentatriacontene***	**C35:2 + *C35:1***	**3477**
n-Pentatriacontane<br>*+ 13-Methylpentatriacontene*	C35+*13MeC35:1*	3502
n-Heptatriacontane<br>*+13-Methylheptatriacontene*	C37+*13MeC37:1*	3698

Supplementary Table S14. Primer information. Primer sequences, amplicon lengths, annealing temperatures (Ta) and source information for the housekeeping gene 28S Ribosomal Protein *RP-S18a* and the immune genes *BGBP*, *PGRP-SC2* and *Def1,* as well as for the *Metarhizium*-specific rRNA gene ITS2.

gene	primer sequence	amplicon length	Ta	source
BGBP	F: 5'-CTGCGCATATCAATTCCCGAC<br>R: 5'-TTCGCTATCTGTCCCGCTTC	101 bp	55°C	Pull et al. 2018[1]
PGRP-SC2	F: 5'-GTGGAGTGGATAACGGCGAA<br>R: 5'-CTATCTCCGGGACAGACGGT	85 bp	55°C	designed based on *Lasius neglectus* cDNA sequence information (Meghan L. Vyleta, AVG, SC unpublished data)
Def1	F: 5'-AGAACACCATCGCGCACGTAG<br>R: 5'-CTGAGAATGCAGTGAGCAGCGC	91 bp	60°C	Konrad et al. 2012[5]
RP-S18a	F: 5'-CGGCTGTATGCTACCACGTA<br>R: 5'-AAGCCTGCTTTCTGAGCCAT	93 bp	55°C	Konrad et al. 2012[5]
ITS2	F: 5'-CCCTGTGGACTTGGTGTTG<br>R: 5'-GCTCCTGTTGCGAGTGTTTT	122 bp	64°C	Giehr et al. 2017[6]

Supplementary Table S15. Ordering details for consumables used in this study.

item	manufacturer	distributor	order number
Triton X-100	Sigma-Aldrich	Merck	X100
sabouraud-4% Dextrose Agar	Sigma-Aldrich	Merck	84088
plastic dish (35 mm x 10 mm)	SPL Life Sciences	Bartelt	11035
plastic dish (35 mm x 10 mm)	Falcon	Bartelt	6.082 394
plastic containers (90 mm x 35 mm)	Licefa GmbH	Licefa GmbH	V2-81
plastic containers (90 mm x 35 mm)	Plastium GmbH	Plastium GmbH	82.000212
D-glucose (U-13C6, 99%)	Cambridge Isotope Laboratories	Eurisotop	CLM-1396-10
1.5 ml screw neck vials 9-425, amber glass	Agilent	Chromatographie Zubehör Trott	45 11 00 012_E
autosampler vial with fixed insert 350 – 10 µl , clear glass	Agilent	Chromatographie Zubehör Trott	451100768
10 ml headspace vial, amber glass	Thermo Fisher Scientific	Chromatographie Zubehör Trott	451801113
10 ml headspace vial, clear glass	Thermo Fisher Scientific	Fisher Scientific GmbH	11506114
20 ml headspace vial, amber glass	Thermo Fisher Scientific	Chromatographie Zubehör Trott	451801211
0.1 ml micro-insert, 30 x 5 x 0.7 mm	Chroma Globe	Bruckner Analysentechnik GmbH	BA10008
one-component-closure-cap 9-425	Agilent	Chromatographie Zubehör Trott	3111C7100
18 mm precision screw caps	Thermo Fisher Scientific	Fisher Scientific GmbH	17303993
screw cap 9-425, Sil / ALU 0.06 mm	Agilent	Chromatographie Zubehör Trott	3111B3085
screw cap 9-425, PTFE/silicone	Agilent	Chromatographie Zubehör Trott	3111S3020
SureSTART™ 18 mm precision screw caps	Thermo Fisher Scientific	Thermo Fisher Scientific	6PMSC18-ST2
n-pentane	Supelco	VWR	1008821000
n-Tetracosane-d50	C/D/N Isotopes	C/D/N Isotopes	D-0883
n-Hexatriacontane-d74	C/D/N Isotopes	C/D/N Isotopes	D-0950
C7 - C40 saturated alkanes standard	Supelco	Merck	49452-U
liner, borosilicate	Joint Analytical Systems	Joint Analytical Systems	JAS-7920-12-5
SPE column, Chromabond, SiOH, 1 ml, 100 mg	Macherey-Nagel	Lactan	N755.1
dimethyl disulfide (DMDS)	Sigma-Aldrich	Merck	471569
iodine, ACS reagent	Sigma-Aldrich	Merck	2307772
diethyl ether	Sigma-Aldrich	Merck	309966
sodium thiosulfate pentahydrate	Sigma-Aldrich	Merck	217247
water, LiChrosolv	Sigma-Aldrich	Merck	1037281002
sodium sulfate, ACS	Sigma-Aldrich	Merck	239313
ASTM 5442 (C12-C60) standard for quantitative linearity	Supelco	Merck	502235
mPAGE® Mini Short Plates	Millipore	Merck	MSHRT
2.8 mm ceramic beads	MO BIO	VWR	MOB013114-325
1 mm zirconia beads	BioSpec Products	Lactan Chemikalien u Laborgeräte	N0381
425-600 µm glass beads	Sigma-Aldrich	Merck	G8772
DNeasy96 Blood and Tissue Kit	Qiagen	Qiagen	69506
Maxwell RSC simply RNA tissue kit	Promega	Promega	AS1340
AllPrep DNA/RNA Mini kit	Qiagen	Qiagen	80204

DNase I	Sigma-Aldrich	Merck	AMPD1-1KT
iScript cDNA synthesis kit	Bio-Rad	Bio-Rad	1708891
2x KAPA SYBR® FAST qPCR Master Mix for Bio-Rad	Roche	Merck	KK4608
primers (Table S14)	Sigma-Aldrich	Merck	

Supplementary Figures

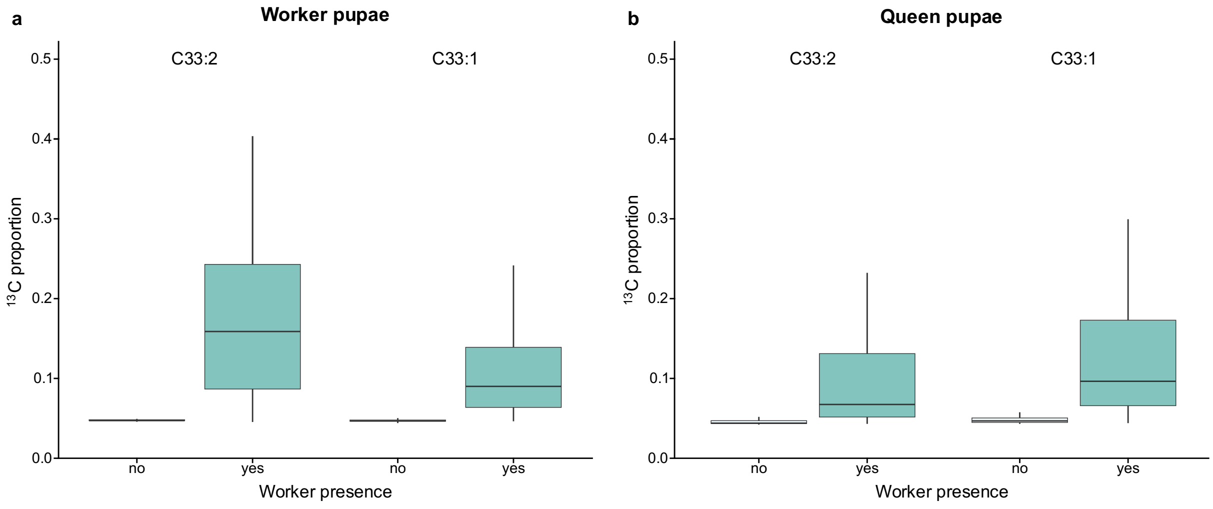

Supplementary Figure S1. ^{13}C transfer from tending workers to worker and queen pupae. The proportion of ^{13}C relative to the total amount of carbon (^{12}C and ^{13}C combined) measured in the two signalling peaks C33:2 and C33:1 of the profiles from of **(a)** worker and **(b)** queen pupae kept in the presence of ^{13}C-enriched workers (green bars; worker pupae n = 190; queen pupae n = 65) vs alone (grey bars; worker pupae n = 133, of which in 5 pupae the C33:2 proportions could not be calculated due to too low peak abundance; queen pupae n = 38). Pupae kept with workers show elevated levels of ^{13}C compared to the natural level of 0.044, whilst the pupae kept alone do not. Bars show means, error bars depict ± sem. Boxes represent interquartile ranges (IQRs) with medians shown as line and whiskers extending to 1.5 x IQR. Source data are provided as Source Data file, raw data deposited under doi.org/10.15479/AT-ISTA-20471.

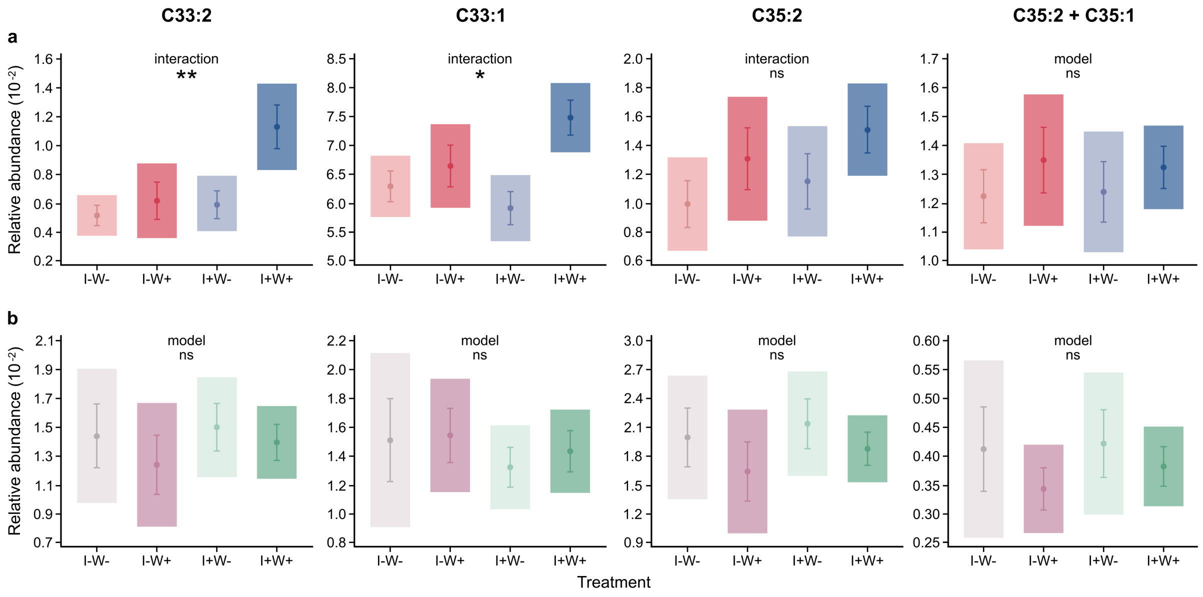

Supplementary Figure S2. CHC peak abundance separately for the four possible candidate peaks identified by Pull et al. 2018[1]. Individual graphs showing the relative pupa-derived abundance of the four candidate CHC peaks, tritriacontadiene (C33:2), tritriacontene, (C33:1), pentatriacontadiene, (C35:2), and co-eluting pentatriacontadiene and pentatriacontene, (C35:2 + C35:1), for **(a)** worker and **(b)** queen pupae that were not infected and kept without workers (I-W-), not infected and kept with workers (I-W+), infected and kept without workers (I+W-) and infected kept with workers (I+W+). Worker pupae (total n = 323) shown in blue for infection and red for control treatment, and queen pupae (total n = 103) in green, resp. purple; absence of workers indicated by pale colours. Dots and bars represent mean ± sem, while shaded area represents 95% confidence intervals of the mean. Statistics (LMs, two-sided p-values after correction for multiple model testing) for worker pupae provided in Table S1, for queen pupae in Table S4. Significant interaction between infection treatment and worker presence shown by ** for p = 0.008 (worker pupae C33:2), * for p = 0.036 (worker pupae C33:1) and ns for p > 0.05 (worker pupae C35:2); model ns represents non-significant overall model (worker pupae C35:2+C35:1, p > 0.05; and all queen pupae peaks: p > 0.05). Source data are provided as Source Data file, raw data deposited under doi.org/10.15479/AT-ISTA-20471.

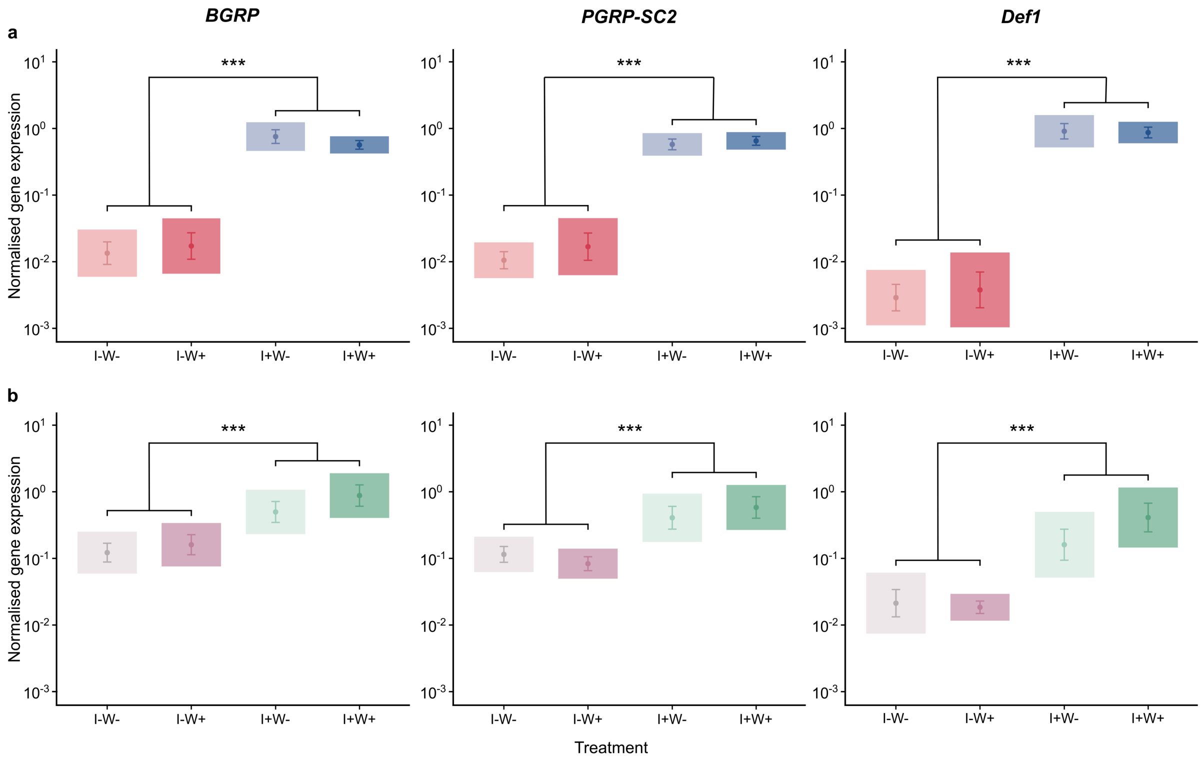

Supplementary Figure S3. Expression patterns of the three individual immune genes. Expression values of the three candidate immune genes β-1,3-glucan binding protein (*BGBP*), peptidoglycan recognition protein SC2 (*PGRP-SC2*) and Defensin (*Def1*), normalised to the expression level of the housekeeping gene 28 S *RP-S18a*, in **(a)** worker pupae and **(b)** queen pupae. Sham treated pupae (I-) in red colour tones, *Metarhizium*-infected (I+) worker pupae in blue and queen pupae in green. Pale colours indicate worker absence (W-), and intense colours worker presence (W+). For all three genes and both castes, we always found the same expression pattern: infected pupae always showed higher immune gene expression (LMs corrected for multiple testing, two-sided p-values of the main effect of infection, worker pupae, n = 117: *BGBP,* p = 1.4×10^{-15}; *PGRP-SC2,* p = 2.2×10^{-16}; *Def1,* p = 2.2×10^{-16} ; queen pupae, n = 62: *BGBP,* p = 0.00013; *PGRP-SC2,* p = 5.3×10^{-5}; *Def1,* p = 2.8×10^{-5}; all depicted by ***), while the presence or absence of workers had no effect (all p > 0.05; Tables S3a, S8a). For visualisation, data were $\log_{10}$-transformed. Source data are provided as Source Data file.

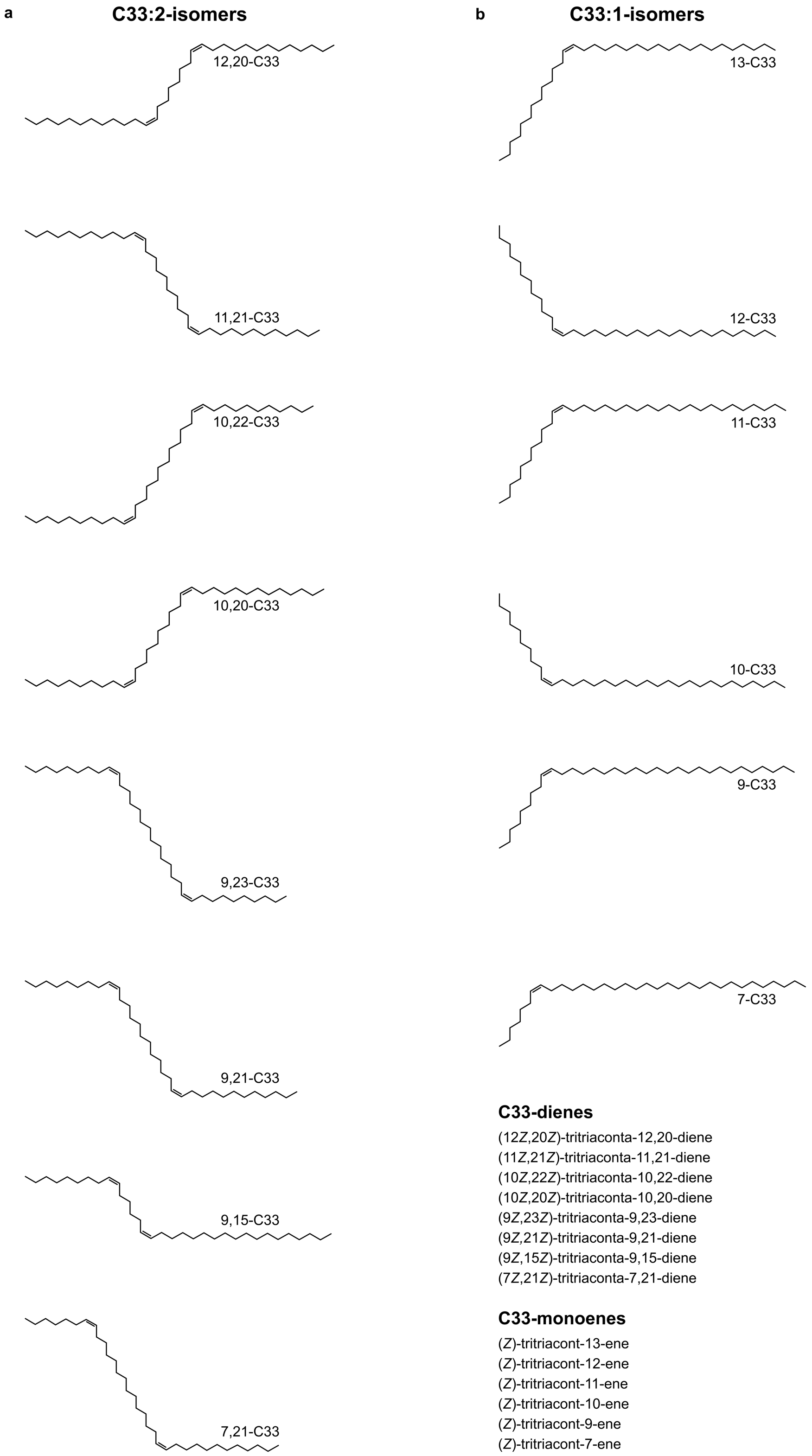

Supplementary Figure S4. Inferred isomeric structures. Structures of the identified (a) C33:2 and (b) C33:1 isomers in their most likely configuration, as (*Z,Z*)-X,Y-C33-dienes and (*Z*)-X-C33-monoenes. The (*Z*)-configuration is inferred, since it is the only so far reported stereoisomeric configuration of naturally-occurring insect CHCs[7], likely driven by their biosynthetic pathways[8], and further corroborated by the retention time overlap of the synthetic (*Z*)-10-C33:1 standard with the C33:1 isomers we identified (Fig. S6), whereas isomers of the alternative (*E*)-configuration would show a shifted retention time range.

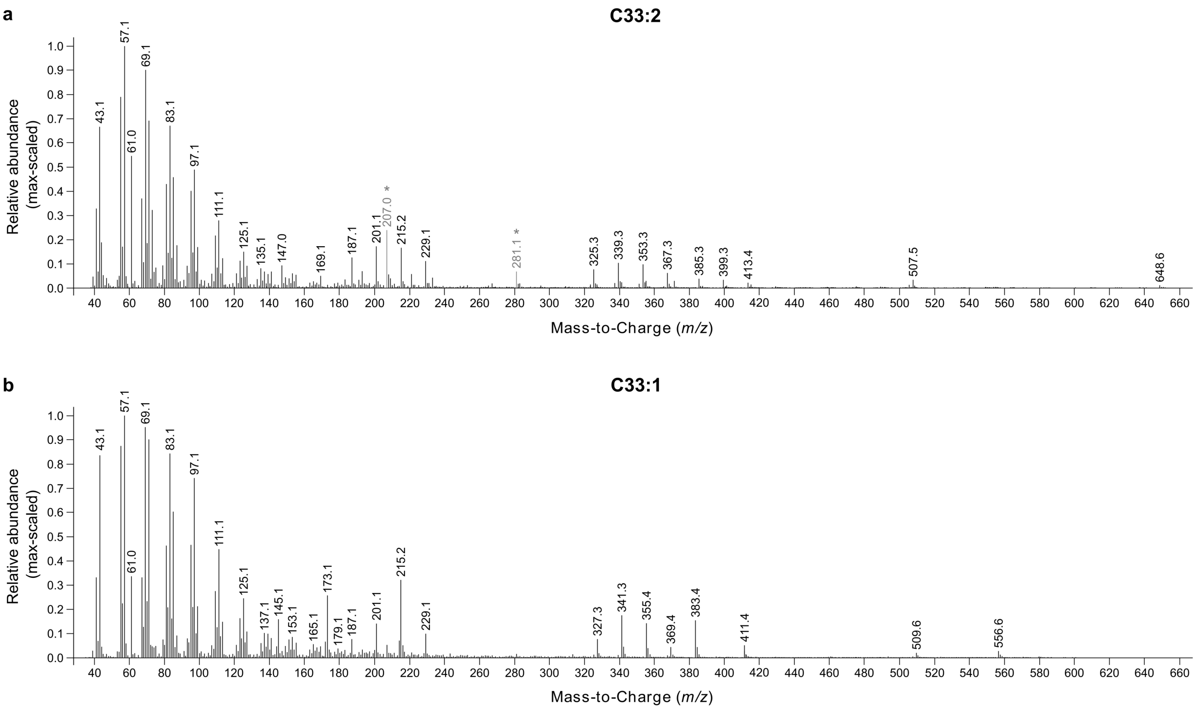

Supplementary Figure S5. Mass spectra of the DMDS-derivatised signal compounds. (a) Mass spectrum averaged across the elution windows of the two dominant C33:2 DMDS-di-adducts 11,21-C33:2 and 10,20-C33:2 (ion pairs with *m/z* 201.1/215.2 and 187.1/229.1 respectively, Table S11). The molecular ion (*m/z* 648.6) and the diagnostic fragment (*m/z* 507.5) were indicative of X,Y-C33-dienes, with additional fragments confirming the di-adducts 11,21-C33:2 and 10,20-C33:2, as detailed in Carlson et al. 1989[2]. Ion traces matching common column bleeding (*m/z* 207, 281) depicted in light grey and with a star *. **(b)** Mass spectrum averaged across the elution windows of all six C33:1-DMDS-mono-adducts (13-, 12-, 11-, 10-, 9- and 7-C33:1; Table S12). The molecular ion (*m/z* 556.6) and the diagnostic fragment (*m/z* 509.6) were indicative of the X-C33-monoenes.

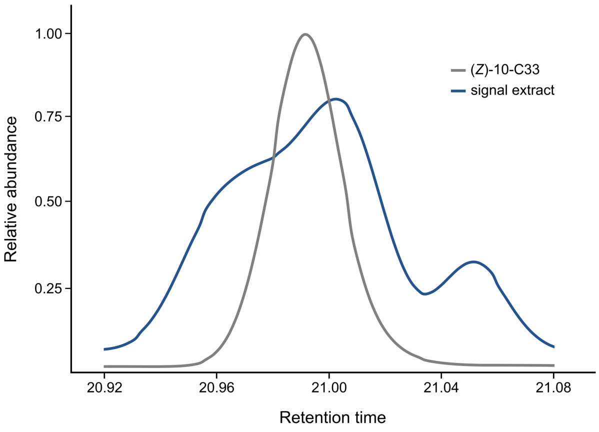

Supplementary Figure S6. Chromatogram overlay of the C33:1 of the signal extract and the synthetic (*Z*)-10-C33:1 standard. Chromatogram of the signal extract (blue), showing the relative abundance (max-scaled) for tritriacontene (C33:1), based on SIM abundances of the molecular ion (*m/z* 462.9). Based on retention time comparisons with a synthetic (*Z*)-10-C33:1 standard (grey) on a DB-5 column, and the compact elution window of the isomers, we infer that the naturally occurring C33:1 isomers (and therefore also the C33:2 isomers eluting closely before) are most likely present in the (*Z*)-configuration. A shift to longer retention times would be expected for (*E*)-isomers, which was not observed. Signal extract obtained from a pool of 20 signalling worker pupae.

Supplementary References

1. Pull, C. D. *et al.* Destructive disinfection of infected brood prevents systemic disease spread in ant colonies. *eLife* **7**, e32073 (2018) https://doi.org/10.7554/eLife.32073.
2. Carlson, D. A., Roan, C. S., Yost, R. A. & Hector, J. Dimethyl disulfide derivatives of long chain alkenes, alkadienes, and alkatrienes for gas chromatography/mass spectrometry. *Anal. Chem.* **61**, 1564–1571 (1989) https://doi.org/10.1021/ac00189a019.
3. Ugelvig, L. V. *et al.* The introduction history of invasive garden ants in Europe: integrating genetic, chemical and behavioural approaches. *BMC Biol.* **6**, 1–14 (2008) https://doi.org/10.1186/1741-7007-6-11.
4. Cremer, S. *et al.* The evolution of invasiveness in garden ants. *PLoS ONE* **3**, e3838 (2008) https://doi.org/10.1371/journal.pone.0003838.
5. Konrad, M. *et al.* Social transfer of pathogenic fungus promotes active immunisation in ant colonies. *PLOS Biol.* **10**, e1001300 (2012) https://doi.org/10.1371/journal.pbio.1001300.
6. Giehr, J., Grasse, A. V., Cremer, S., Heinze, J. & Schrempf, A. Ant queens increase their reproductive efforts after pathogen infection. *Roy. Soc. Open Sci.* **4**, 170547 (2017) https://doi.org/10.1098/rsos.170547.
7. Menzel, F. *et al.* Communication versus waterproofing: the physics of insect cuticular hydrocarbons. *J. Exp. Biol.* **222**, jeb210807 (2019) https://doi.org/10.1242/jeb.210807.
8. Morgan, E. D. *Biosynthesis in Insects*. (Royal Society of Chemistry, Cambridge, UK, 2010).
