# Supplementary material for: Altruistic disease signalling in ant colonies
Source: Nat Commun. 2025 Dec 2;16:10511. doi: 10.1038/s41467-025-66175-z (PMC12672639; doi:10.1038/s41467-025-66175-z)
Supplement: Supplementary file 2 — Reporting Summary [file 41467_2025_66175_MOESM2_ESM.pdf]

nature portfolio

Corresponding author(s): Erika H. Dawson and Sylvia Cremer

Last updated by author(s): 2025/10/24

Reporting Summary

Nature Portfolio wishes to improve the reproducibility of the work that we publish. This form provides structure for consistency and transparency in reporting. For further information on Nature Portfolio policies, see our Editorial Policies and the Editorial Policy Checklist.

Statistics

For all statistical analyses, confirm that the following items are present in the figure legend, table legend, main text, or Methods section.

n/a	Confirmed	
☐	☒	The exact sample size (*n*) for each experimental group/condition, given as a discrete number and unit of measurement
☐	☒	A statement on whether measurements were taken from distinct samples or whether the same sample was measured repeatedly
☐	☒	The statistical test(s) used AND whether they are one- or two-sided<br>*Only common tests should be described solely by name; describe more complex techniques in the Methods section.*
☐	☒	A description of all covariates tested
☐	☒	A description of any assumptions or corrections, such as tests of normality and adjustment for multiple comparisons
☐	☒	A full description of the statistical parameters including central tendency (e.g. means) or other basic estimates (e.g. regression coefficient) AND variation (e.g. standard deviation) or associated estimates of uncertainty (e.g. confidence intervals)
☐	☒	For null hypothesis testing, the test statistic (e.g. *F*, *t*, *r*) with confidence intervals, effect sizes, degrees of freedom and *P* value noted<br>*Give P values as exact values whenever suitable.*
☒	☐	For Bayesian analysis, information on the choice of priors and Markov chain Monte Carlo settings
☒	☐	For hierarchical and complex designs, identification of the appropriate level for tests and full reporting of outcomes
☐	☒	Estimates of effect sizes (e.g. Cohen's *d*, Pearson's *r*), indicating how they were calculated

Our web collection on statistics for biologists contains articles on many of the points above.

Software and code

Policy information about availability of computer code

Data collection	Software used for real-time PCR: Bio-Rad CFX Manager software.<br>Software for gas chromatography-mass spectrometry: CHRONOS 4.2 software, Axel Semrau and MassHunter Workstation, Data Acquisition software B.07.01 and Qualitative and Quantitative Analysis: Agilent Technologies
Data analysis	Statistics: R version 4.3.2 and R studio Version 2023.12.1.402, with packages 'bestNormalize' (vs 1.9.1), 'lme4' (vs 1.1.36), 'survival' (vs 3.5.7), 'emmeans' (vs 1.10.0), 'Rstatix' (vs 0.7.2), 'lmtest' (vs 0.9.40).<br>Figures: R version 4.5.0 and R studio Version 2023.06.0.421 with packages 'ggplot2' (vs 3.5.2), dplyr (vs 1.1.4), ggpubr (vs 0.6.0) and Rmisc (vs 1.5.1).

For manuscripts utilizing custom algorithms or software that are central to the research but not yet described in published literature, software must be made available to editors and reviewers. We strongly encourage code deposition in a community repository (e.g. GitHub). See the Nature Portfolio guidelines for submitting code & software for further information.

Data

Policy information about availability of data

All manuscripts must include a data availability statement. This statement should provide the following information, where applicable:

- Accession codes, unique identifiers, or web links for publicly available datasets
- A description of any restrictions on data availability
- For clinical datasets or third party data, please ensure that the statement adheres to our policy

As reported in the Data Availability Statement, the data generated in this study are provided in the Source Data file. Additional raw data of the integrated 12C and 13C areas of all samples, as well as the mass spectra of the native CHCs (Table S13) have been deposited in the ISTA Research Explorer (ISTA REx) repository under accession code doi.org/10.15479/AT-ISTA-20471.

Research involving human participants, their data, or biological material

Policy information about studies with human participants or human data. See also policy information about sex, gender (identity/presentation), and sexual orientation and race, ethnicity and racism.

Reporting on sex and gender	N/A
Reporting on race, ethnicity, or other socially relevant groupings	N/A
Population characteristics	N/A
Recruitment	N/A
Ethics oversight	N/A

Note that full information on the approval of the study protocol must also be provided in the manuscript.

Field-specific reporting

Please select the one below that is the best fit for your research. If you are not sure, read the appropriate sections before making your selection.

- [ ] Life sciences
- [ ] Behavioural & social sciences
- [x] Ecological, evolutionary & environmental sciences

For a reference copy of the document with all sections, see nature.com/documents/nr-reporting-summary-flat.pdf

Ecological, evolutionary & environmental sciences study design

All studies must disclose on these points even when the disclosure is negative.

Study description

Worker and queen pupae of the ant Lasius neglectus were either infected with the fungal pathogen Metarhizium brunneum or treated with a control solution. After an initial solitary phase that allowed for infection establishment, the pupae were either kept alone or put with two tending workers, resulting in four treatment groups: ‘control pupae without workers (I-W-)’, ‘control pupae with workers (I-W+)’, ‘infected pupae without workers (I+W-)’ and ‘infected pupae with workers (I+W+)’. From each treatment, we sampled pupae over a period of 42 h after setting them up with the workers, at seven time points, in intervals of six hours (i.e. at 6, 12, 18, 24, 30, 36 and 42 h) to analyse (i) the pupal cuticular hydrocarbons (CHCs) by identification of CHC compounds and their quantities using gas chromatography-mass spectrometry (GC-MS) and (ii) the fungal load of each pupa using quantitative real-time PCR (qPCR). For both, worker and queen pupae, we determined if infection treatment and worker presence affected the relative abundances of four previously identified CHC peaks (Pull et al 2018; linear model testing the effect of the interaction: infection treatment * worker presence), if unpacking differed between infected and control pupae (Cox proportional-hazards regression analyses) and, how fungal infection progressed over the course of the experiment (linear model testing the effect of infection period on infection load). We employed the same experimental design to quantify pupal gene expression of three candidate immune genes (BGBP, PGRP-SC2 and Def1) in both pupal castes for the four treatment groups, again using qPCR (linear model testing for the main effects of infection treatment and worker presence after ns interaction). To test whether the altered CHC profile of I+W+ worker pupae was sufficient to induce worker unpacking, we created CHC extract from signalling (I+W+) and non-signalling control (I-W+) worker pupae. We applied the extracts to healthy worker and queen pupae to observe if application of signal extract elicited higher unpacking than the control extract (binomial model with one-sided hypothesis testing). We further determined the chemical nature of the signalling compounds by identifying their isomeric composition following DMDS-derivatisation.

Research sample

For our study, we used garden ants Lasius and their natural pathogen, the common fungal entomopathogen Metarhizium, which is a well-established model system for the study of social immunity in ants (see e.g. Cremer et al. 2018 Annu Rev Entomol). As the pathogen, we used M. brunneum strain Ma275 (KVL 04-57, obtained from N.V. Meyling and J. Eilenberg from the University of Copenhagen, Denmark). As the host, we used the invasive garden ant, Lasius neglectus, collected in Jena, Germany (N 50° 55’ 54.599” E 11°35’ 8.401”) every spring and/or summer from 2018 to 2024 except for 2020, and in Seva, Spain (N 41°48’ 32.699" E 2° 15' 42.3") during late spring or early summers in 2016, 2018, 2023 and 2024 (for compliance with the Nagoya protocol for Access and Benefit Sharing see below). The field-collected insects were brought back to the laboratory and reared in stock colonies until use in the experiments. Worker pupae used in the experiments originated from both populations, queen pupae only from the Jena

population. All experimental work was performed in the laboratory.

Sampling strategy

We studied the effect of infection status and worker presence on the CHC profile and the infection progression of a total of 323 worker pupae (64 I-W-, 57 I-W+, 69 I+W-, 133 I+W+), and the pupal unpacking in the W+ groups. The pupae originated equally from the two populations (Jena, n = 168 and Seva, n = 155), which represent independent introductions, resp. supercolonies of L. neglectus (Pull et al 2018 eLife had already established generality across populations of L. neglectus). Queen pupae are only produced at a limited number during a short period of the year (shortly before the mating flight in the summer), and could only be used in the experiments from the Jena population. A total of 103 queen pupae (19 I-W-,20 I-W+, 19 I+W-, 45 I+W+) were equally analysed as the worker pupae. In addition, several queen pupae were produced by the ants in the laboratory in 2021 from the 2018 Jena collection. These pupae were not used in the experiment, but to quantify exposure dose (i.e. the baseline fungal load immediately after spore application). All pupae in the experiments were individually exposed to the pathogenic fungus or a pathogen-free sham treatment and kept isolated for an incubation period. The infected or control pupae were then either kept alone or with two workers, so that 510 workers accompanied the pupae in our experiment (380 workers with worker pupae and 130 with queen pupae). All 426 pupae were individually analysed for their 12C and 13C composition of the CHCs in their chemical bouquet (18 analysable peaks), and the same was done for the 255 pools of the two workers per replicate, leading to a total of 681 GC-MS samples. For fungal load analysis, one infected worker pupa was lost and three control samples could not be analysed, leading to a total of 422 qPCRs. Our sample sizes allowed for a time-resolved Cox Proportional Hazard Model for worker unpacking behaviour across our sampling scheme of freezing pupae every 6 h over a period of 42 h. This scheme was chosen to make sure that the unpacked pupae were not yet destructed by the workers at the time of sampling, but still retained their capacity for active modulation of their CHCs. For the chemical, infection load and immune gene expression analyses, we ensured high statistical power by combining the sampling time points 6 - 12 h to an early, 18 - 24 h to a middle and 30 - 42 h to a late 'infection period'. For the immune gene expression we analysed a total of 117 worker pupae (20 I-W-, 20 I-W, 19 I+W-, 58 I+W+, +) and 62 queen pupae (11 I-W-, 14 I-W+, 17 I+W-, 20 I+W+), with all pupae originating from the Jena population. To create signal and non-signal extract for the bioassay, we extracted the CHCs of further 343 signalling worker pupae (I+W+) and of 338 non-signalling (I-W+) worker pupae, obtained from both populations. We then observed whether workers unpacked healthy pupae more when they had received the signal extract (76 worker pupae with signal and 64 with non-signal extract; 35 queen pupae with signal and 38 with non-signal extract). For CHC isomer characterisation, we extracted an additional 20 signalling and 20 non-signalling worker pupae.

Data collection

Worker feeding and pupal experiments for the CHC profiles and infection progression were carried out by EHD & NK (worker pupae) and JR, AVG & FS (queen pupae). Experiments for the immune gene expression were performed by JR, AVG & FS (worker pupae) and by AVG, FB & SC (queen pupae). Pupal experiments for the signal and non-signal extract generation were run by AVG, FB, HR, HL, FS and SC. Extractions were done by LL. Extract applications were carried out by LL and FB. Chemical data generation by GC-MS was carried out by NK and MH, and DMDS-derivatisation was performed by MH. Fungal quantification and immune gene expression by qPCR were performed by AVG.

Timing and spatial scale

Worker pupae were exposed to fungal resp. control suspensions and left alone for three days prior to the start of experiments to allow an infection to develop without interference from workers. After adding the workers, pupae were sampled every six hours for 42 hours to ensure that pupae were not destructed by the workers and could subsequently be analysed for their chemical profiles, pathogen loads and immune gene expression. The worker pupae experiment for CHCs and infection progression was run in 2019, followed by GC-MS analysis of worker pupae and accompanying workers in 2020 and fungal load quantification in 2022/23. Queen pupae were collected from the field in Jena in 2021, and used directly thereafter. Analysis of the queen pupae and accompanying workers by GC-MS was performed in 2021/22 and fungal load qPCR in 2022/23. The worker pupae experiment for gene expression analysis was performed in 2021. Most of the samples were analysed in the qPCR in 2021, except for several sham-treated pupae that were analysed in 2024 only. The queen pupae experiment for gene expression analysis was set up after collection from the field in Jena in 2024 and samples were run in the qPCR later in 2024. The extract generation and application experiments were carried out in 2024. DMDS-derivatisation and following isomer characterisation were performed in 2024/25.

Data exclusions

The above-given sample sizes represent the final sample sizes of our experiments. For the CHC and infection progression experiment, we had originally set up 406 replicates, yet excluded 27 samples as the pupae got harmed or died, 18 in which one or both workers died during the experiment and three where the pupae got lost during sampling, leading to 358 replicates. 35 of them did not fulfil our criteria to allow a 12C to 13C comparison in their chemical profiles, and were hence not included in the analysis, leading to a final sample size of 323 worker pupae. For the queen pupae, only one pupa did not fulfil the chemical criteria, so that 103 (out of 104) could be analysed. Chemical exclusion was performed since accurate assessment of their pupa-produced CHC quantity was not possible for all compounds. This was either because – likely due to natural variation or measurement inaccuracies at low compound abundances – the 13C proportion of some of their compounds had a higher value than that of accompanying workers (worker pupae experiment: n=21; queen pupae experiment: n=0) or as their 13C was below detection threshold in some compounds that were typically overall of low quantity (worker pupae experiment: n=14 pupae; queen pupae experiment: n=1). From our 202 infected worker pupae, we lost one sample after GC-MS and before qPCR analysis, leading to a final sample size of fungal load quantification of 201 worker and all 64 infected queen pupae (265 samples). In addition, we also ran the sham-treated control pupae to confirm absence of fungal infection. Three of the control pupae could not be analysed in the qPCR for technical reasons. In the immune gene expression experiment, 12 queen pupae developed mould or died, three queen pupae were not of the right developmental stage, and the RNA of nine worker pupae was of poor quality leading to a final sample size of 117 worker pupae and 62 queen pupae. In the bioassay, two worker pupae had to be excluded after application of the signalling extract, due to mechanical damage.

Reproducibility

Whereas the bioassay was run in seven blocks (three for the worker pupae and four for the queen pupae), our experiments for CHCs and infection progression, resp. immune gene expression, were each run in a single block and reproducibility was obtained by simultaneous replication. For the worker pupae, the CHC and infection experiment could be run for two ant populations. In the qPCR, samples as well as standards and controls were run in triplicates. This study is based on a previous study by Pull et al. 2018 eLife, which had identified four candidate peaks in worker-unpacked worker pupae, two of which were immune-associated, and had established generality across the two populations of Jena and Seva. Our current work confirmed the relevance of the CHCs identified by this earlier study, and highlighted the particular role of the immune-associated CHCs as chemical signals. This reveals high robustness even across years, in which ants were collected from the field, as well as different experimenters.

Randomization

When setting up the experiments, pupae as well as workers were picked randomly from inside the brood chambers in the laboratory stock colonies. Each pupa was then randomly assigned to an infection treatment group (Metarhizium brunneum in Triton X, or the Triton X sham control). After the three days incubation period, pupae from both treatment groups were then randomly selected to

be put in experimental dishes with or without two workers. For the GC-MS, the extracts from the different treatment groups were run in a randomised manner, intermingled with blank runs (containing only n-pentane), and handling controls. In the qPCR, samples were run intermingled with water controls and standards. In the bioassay, the different batches were intermingled.

Blinding	Our experimental procedures were inherently implemented in a bias-free manner, as (i) experimental sample collection involved freezing of pupae at predefined sampling times, (ii) determination of the unpacking status is free of interpretation since pupae are either enclosed in their cocoons or had been unpacked by the workers, (iii) chemical and molecular analyses are not vulnerable to subjectivity since neither the chemical signal nor pathogen load is detectable by the experimenter, and quantification of CHCs is based on comparison to standards for each run in the GC-MS and quantification of fungal load in the qPCR equally determined by use of standards. In the bioassay, extract information was not accessible for the observer.

Did the study involve field work? ☐ Yes ☒ No

Reporting for specific materials, systems and methods

We require information from authors about some types of materials, experimental systems and methods used in many studies. Here, indicate whether each material, system or method listed is relevant to your study. If you are not sure if a list item applies to your research, read the appropriate section before selecting a response.

Materials & experimental systems

n/a	Involved in the study
☒	☐ Antibodies
☒	☐ Eukaryotic cell lines
☒	☐ Palaeontology and archaeology
☐	☒ Animals and other organisms
☒	☐ Clinical data
☒	☐ Dual use research of concern
☒	☐ Plants

Methods

n/a	Involved in the study
☒	☐ ChIP-seq
☒	☐ Flow cytometry
☒	☐ MRI-based neuroimaging

Animals and other research organisms

Policy information about studies involving animals; ARRIVE guidelines recommended for reporting animal research, and Sex and Gender in Research

Laboratory animals	The insects (Lasius neglectus ants) used in our experiments were collected in the field as detailed below and reared in the laboratory under a temperature and light cycle reflecting natural seasons, until use in the experiments. For the experiments, we sampled workers from chambers inside the nest to avoid old foragers. We also did not sample freshly emerged individuals (callows, which can be distinguished by their pale colour due to missing melanisation and sclerotisation of the cuticle). Only pupae of a standardized developmental stage (white pupae with black eyes) were sampled. In the worker pupae, this information was obtained by visual inspection of each pupa through the cocoon using a stereo microscope. In the queen pupae, the cocoon is visually not transmissive, but pupal stage could be assessed by opening the cocoons of some pupae of the colony, as they develop in same-age cohorts.
Wild animals	We used workers and queen and worker pupae of the invasive garden ant, Lasius neglectus, in our experiments. We collected the ants from their nests in the soil in their introduced populations in Jena, Germany (N 50° 55’ 54.599” E 11°35’ 8.401”) between 2018 and 2024, and in Seva, Spain (N 41°48’ 32.699" E 2° 15' 42.3") between 2016 and 2024. From both populations, we collected queens, workers and worker brood. Queen brood in the early enough pupal stage could only be obtained from Jena in 2021 and 2024 (in addition, several queen pupae developed in 2021 in the laboratory from ants collected in Jena in 2018, yet these were only used for exposure dose determination and not in the main experiments). After field collections, ants were transported back to the laboratory in plastic boxes, and reared in the laboratory until their use in the experiments, which all occurred under standardised conditions in the laboratory.
Reporting on sex	As ants are Hymenoptera, all worker and queen ants are female.
Field-collected samples	After collection, ants were reared until use in the experiments in incubators reflecting the yearly temperature and a circadian day/night light cycle. For the experiments, the pupae and workers were taken from their stock colony when in summer conditions, and placed in experimental dishes with humidified plastered floor in a temperature- and humidity-controlled climate room at 23 °C and 65% RH.
Ethics oversight	We used ants (insects, invertebrates) as our study animals. In particular, we used the invasive garden ant, Lasius neglectus. Collection of this invasive, unprotected species from the field was in compliance with international regulations, such as the Convention on Biological Diversity and the Nagoya Protocol on Access and Benefit-Sharing (ABS) and was granted by the Spanish Ministry of Agriculture, Fisheries and Environment (permit no. ESNC12 and ESNC126) and Catalonian authorities (permit no. SF/0558-0561 and EPI 92-2023). The collection in Germany did not require any specific permits. Transport to and rearing of the ants in the laboratory, as well as all experimental work followed European and Austrian law and institutional ethical guidelines of the Institute of Science and Technology Austria (ISTA).

Note that full information on the approval of the study protocol must also be provided in the manuscript.
